# Indications and pitfalls of immunohistochemistry in head and neck cancer

**DOI:** 10.5935/1808-8694.20130013

**Published:** 2015-10-14

**Authors:** Décio de Natale Caly, Acklei Viana, Abrão Rapoport, Rogério Aparecido Dedivitis, Otávio Alberto Curioni, Cláudio Roberto Cernea, Lenine Garcia Brandão

**Affiliations:** PhD in Pathology from A.C. Camargo Hospital, São Paulo (Pathologist at the Heliópolis Hospital, São Paulo); Resident Physician in the Department of Head and Neck Surgery and Otorhinolaryngology at the Heliópolis Hospital, São Paulo; Associate Professor in the Department of Surgery at the Medical School of the University of São Paulo (Technical Director in the Department of Health at the Heliópolis Hospital, São Paulo); Associate Professor. Supervisor of the Larynx Group in the Department of Head and Neck Surgery of the University Hospital of the Medical School of the University of São Paulo (MD); PhD in Pathology from the Medical School of the University of São Paulo (Head of the Department of Head and Neck Surgery and Otorhinolaryngology at the Heliópolis Hospital, São Paulo); Associate Professor in the Department of Head and Neck Surgery at the Medical School of the University of São Paulo, São Paulo; Professor in the Department of Head and Neck Surgery at the Medical School of the University of São Paulo, São Paulo. Department of Head and Neck Surgery and Otorhinolaryngology at the Heliópolis Hospital, São Paulo and Department of Head and Neck Surgery at the Medical School of the University of São Paulo, São Paulo

**Keywords:** carcinoma, squamous cell, head and neck neoplasms, immunohistochemistry, lymphoma, sarcoma

## Abstract

Immunohistochemistry (IHC) has been employed in the differential diagnosis of tumors.

**Objective:**

To assess the use of IHC in cases of head and neck tumor.

**Method:**

This is a retrospective study of the cases included in the Cancer Registry of the institution.

**Results:**

IHC was used in 76 (11%) of 704 pathology tests. Most cases were carcinomas (85.80%), and 83.66% of them were squamous cell carcinomas. All tests were done with diagnostic purposes. The most frequently used antibodies were 34BE12 (37.18%), AE1/AE3 (35.9%), 35BH11 (28.21%), CD45 (25.64%), CD20 (24.36%), CD30 (24.36%), CK7 (23.08%) and CD3 (23.08%).

**Conclusions:**

IHC was used in 10.67% of the head and neck tumor cases submitted to pathology testing, mostly for carcinoma (5.26%). In the determination of squamous cell carcinoma, IHC accounted for 18.42% of all tumors.

## INTRODUCTION

Macro and microscopic alterations lie in the basis of pathology diagnostics, and microscopic morphology plays a fundamental role in the interpretation of histogenesis, classification, and prognostic assessment of tumors. However, in the 1970s more elaborate techniques gained significant ground in aiding in the diagnosis of cases in which the assessment produced from the use of the usual histochemical stains was not conclusive. Immunohistochemistry (IHC) protocols were developed in this setting, using antibodies tagged with chromogens to identify specific markers. In these protocols, antigen-antibody reactions using non-fluorescent chromogens are analyzed in an optical microscope. IHC is performed in paraffin-embedded specimens sliced on a microtome and submitted to treatments for antigen retrieval, processed with immunoperoxidase and development reagents^1^^,^^2^. The ease of handling paraffin-embedded specimens has granted significant popularity to IHC in pathology labs.

Despite the relevant contributions offered by IHC, the assessment of the specimens and the determination of which panel to use is made only after careful analysis by the pathologist through traditional pathology testing protocols.

The main indications for IHC are: definition of histogenesis; differential diagnosis between reactive and neoplastic states; etiologic diagnosis of infectious diseases; determination of prognostic factors; determination of target therapy sites; location of primary tumors in malignant disease; determination of specific products (such as hormones and proteins); and cancer subtyping.

In the definition of histogenesis, assessment is carried out to determine the tissue and/or cell origin of a specific cell population. In the case of neoplasms, IHC provides substantial help in defining whether the tumor derives from epithelial, mesenchymal, hematopoietic, or nervous tissue.

The criteria to define whether a tumor is benign or malignant are still strongly founded in morphology. Macroscopically, features such as infiltrative growth pattern, borders of the tumor, absence of a tumor capsule, growth rate, necrosis, and hemorrhage are considered. Microscopically, cell count and anaplasia (cell and nucleus pleomorphism, atypical mitosis, and nuclear-cytoplasmic ratio) are analyzed.

It is important that these ideas be considered, as when IHC is used to determine histogenesis, the tests yield positive results for the same markers based on tissue origin and not on whether the tumor is benign or malignant, i.e., normal malpighian (squamous) epithelium will be marked the same way as squamous cell carcinoma.

Histological classification is based on the criteria defined by Broders, in which cell differentiation and keratinization of tumor cells are categorized into four grades of final differentiation^3^. Squamous cell carcinoma (SCC) may present the following variants, according to the classification of the World Health Organization: verrucous, basaloid, papillary, spindle-cell, acantholytic, adenosqua-mous, and cuniculatum^4^.

However, neoplasms other than SCC may involve the head and neck area, including undifferentiated carcinomas, metastasis, neuroendocrine carcinomas, sarcomas, lymphomas, etc.

In this setting IHC is required to produce diagnosis and subtype tumors. Despite the routine use of IHC and its ample availability for clinical and surgical personnel, it is worthwhile discussing its use and precise indications in diagnostics, prognostics, the possible impacts it may have upon therapy, and the technique's limitations. Thus, this paper aimed to assess the use of IHC in cases of head and neck cancer.

## METHOD

This retrospective study looked into the pathology test results of the Cancer Registry of the institution dated from April 2009 to February 2012, and included cases of the hospital's Head and Neck Service.

Enrollment criteria included: subjects diagnosed and treated at the institution with cases entered into the Hospital Cancer Registry; pathology tests filed in electronic format in the Hospital Cancer Registry. Exclusion criteria: lack of IHC testing support; thyroid tumors; basal-cell carcinoma of the skin and cutaneous attachments; and odontogenic tumors.

The study included cases processed by IHC protocols, considering the incidence rates of various tumor types - carcinomas, sarcomas, and lymphomas - and utilized panels.

Descriptive analysis was performed based on absolute and relative frequencies.

## RESULTS

A grand total of 677 pathology test results were selected; IHC was not performed in 605 (89.4%) cases; seventy-two (10.6%) cases had IHC test results.

Considering the split of tumor types, carcinomas accounted for 94.69% of the cases, followed by lymphomas (3.84%). IHC panels for diagnostic purposes were used in 10.64% of the cases, 5.47% of which were epithelial tumors. IHC was done in all cases of lymphoma and sarcoma (Table 1). In terms of histological subtypes, SCC accounted for most cases (87%). The other subtypes are listed on Table 2.

**Table 1:** Distribution of diagnoses.

	No IHC		IHC		Total	
	Cases	Percent	Cases	Percent	Cases	Percent
Carcinoma	604	89.22%	37	5.47%	641	94.68%
Lymphoma	0	0.00%	26	3.84%	26	3.84%
Sarcoma	0	0.00%	7	1.03%	7	1.03%
Melanoma	1	0.15%	2	0.30%	3	0.44%
Total	605	89.36%	72	10.64%	677	100.00%

**Table 2:** Distribution of histological tumor subtypes.

	No IHC		IHC		Total	
	Cases	Percent	Cases	Percent	Cases	Percent
Squamous cell carcinoma	575	84.93%	14	2.07%	589	87.00%
Undifferentiated carcinoma	16	2.36%	6	0.89%	22	3.25%
Node metastasis by adenocarcinoma	8	1.18%	9	1.33%	17	2.51%
Mucoepidermoid carcinoma	1	0.15%	1	0.15%	2	0.30%
Adenoid cystic carcinoma	1	0.15%	1	0.15%	2	0.30%
Parotid adenocarcinoma	1	0.15%	2	0.30%	3	0.44%
Nasopharyngeal carcinoma	0	0.00%	1	0.15%	1	0.15%
Small-cell carcinoma	1	0.15%	1	0.15%	2	0.30%
Palate adenocarcinoma	0	0.00%	1	0.15%	1	0.15%
Neuroendocrine carcinoma	1	0.15%	1	0.15%	2	0.30%
Pleomorphic sarcoma	0	0.00%	2	0.30%	2	0.30%
Undifferentiated sarcoma	0	0.00%	1	0.15%	1	0.15%
Fibromyxoid sarcoma	0	0.00%	1	0.15%	1	0.15%
Chondrosarcoma	0	0.00%	1	0.15%	1	0.15%
Leiomyosarcoma	0	0.00%	1	0.15%	1	0.15%
Angiosarcoma	0	0.00%	1	0.15%	1	0.15%
Hodgkin's lymphoma	0	0.00%	7	1.03%	7	1.03%
Diffuse large B-cell non-Hodgkin's lymphoma	0	0.00%	14	2.07%	14	2.07%
Mantle cell lymphoma	0	0.00%	1	0.15%	1	0.15%
Plasmablastic lymphoma	0	0.00%	2	0.30%	2	0.30%
Anaplastic large cell lymphoma	0	0.00%	1	0.15%	1	0.15%
Follicular lymphoma	0	0.00%	1	0.15%	1	0.15%
Melanoma	0	0.15%	2	0.30%	3	0.44%
Total	605	89.36%	72	10.64%	677	100.00%

In the 72 cases submitted to immunohistochemistry testing, SCC (19.44%) and diffuse large cell lymphomas tied for first (19,44%), followed by node metastasis (12.50%), Hodgkin's lymphoma (9.72%), and undifferentiated carcinoma (8.33%) (Table 3).

**Table 3:** Distribution of diagnoses obtained from immunohistochemistry testing.

	Cases	Percent
Squamous cell carcinoma	14	19.44%
Undifferentiated carcinoma	6	8.33%
Node metastasis by adenocarcinoma	9	12.50%
Mucoepidermoid carcinoma	1	1.39%
Adenoid cystic carcinoma	1	1.39%
Parotid adenocarcinoma	2	2.78%
Nasopharyngeal carcinoma	1	1.39%
Small-cell carcinoma	1	1.39%
Palate adenocarcinoma	1	1.39%
Neuroendocrine carcinoma	1	1.39%
Pleomorphic sarcoma	2	2.78%
Undifferentiated sarcoma	1	1.39%
Fibromyxoid sarcoma	1	1.39%
Chondrosarcoma	1	1.39%
Leiomyosarcoma	1	1.39%
Angiosarcoma	1	1.39%
Hodgkin's lymphoma	7	9.72%
Diffuse large B-cell non-Hodgkin's lymphoma	14	19.44%
Mantle cell lymphoma	1	1.39%
Plasmablastic lymphoma	2	2.78%
Anaplastic large cell lymphoma	1	1.39%
Follicular lymphoma	1	1.39%
Melanoma	2	2.78%
Total	72	100.00%

When incidence rates within specific tumor groups (carcinoma, lymphoma, sarcoma) were compared for diagnosed subtypes for which IHC was done, the highest rates in the carcinoma group went to SCC, with 37.84%; in the lymphoma group, diffuse large B-cell lymphoma ranked first, with 53.85%; and in the sarcoma group, pleomorphic sarcoma was the most commonly observed tumor type, with 28.57% (Table 4, Table 5, Table 6).

**Table 4:** Distribution of carcinomas tested with immunohistochemistry based on subtypes.

	Cases	Percent
Squamous cell carcinoma	14	37.84%
Undifferentiated carcinoma	6	16.22%
Node metastasis by adenocarcinoma	9	24.32%
Mucoepidermoid carcinoma	1	2.70%
Adenoid cystic carcinoma	1	2.70%
Parotid adenocarcinoma	2	5.41%
Nasopharyngeal carcinoma	1	2.70%
Small-cell carcinoma	1	2.70%
Palate adenocarcinoma	1	2.70%
Neuroendocrine carcinoma	1	2.70%
Total	37	100.00%

**Table 5:** Distribution of sarcomas submitted to immunohistochemistry testing.

	Cases	Percent
Pleomorphic sarcoma	2	28.57%
Undifferentiated sarcoma	1	14.29%
Fibromyxoid sarcoma	1	14.29%
Chondrosarcoma	1	14.29%
Leiomyosarcoma	1	14.29%
Angiosarcoma	1	14.29%
Total	7	100.00%

**Table 6:** Distribution of lymphomas submitted to immunohistochemistry testing.

	Cases	Percent
Hodgkin's lymphoma	7	26.92%
Diffuse large B-cell non-Hodgkin's lymphoma	14	53.85%
Mantle cell lymphoma	1	3.85%
Plasmablastic lymphoma	2	7.69%
Anaplastic large cell lymphoma	1	3.85%
Follicular lymphoma	1	3.85%
Total	26	100.00%

The 76 immunohistochemistry tests were done with diagnostic purposes. Considering the chosen IHC panels and antibodies, 34BE12 (37.18%), AE1/AE3 (35.90%), 35BH11 (28.21%), CD45 (25.64%), CD20 (24.36%), CD30 (24.36%), CK7 (23.08%) and CD3 (23.08%) were the most frequently used (Table 7).

**Table 7:** Distribution of immunohistochemistry markers.

Marker	Percent
34BE12	37.18%
AE1/AE3	35.90%
35BH11	28.21%
CD45	25.64%
CD20	24.36%
CD30	24.36%
CK7	23.08%
CD3	23.08%
P-S100	19.23%
CD15	17.95%
CK20	16.67%
P63	16.67%
Vimentin	14.10%
Ki67	14.10%
AML 1-4	11.54%
HMB45	11.54%
CD10	11.54%
BCL2	11.54%
Melan A	8.97%
BLC6	8.97%
CK19	7.69%
Chromogranin	7.69%
EMA	7.69%
Desmin	7.69%
Synaptophysin	6.41%
HHF35	6.41%
CD79a	6.41%
Cyclin D1	6.41%
CK5/6	2.56%
CD5	2.56%
Thyroglobulin	2.56%
Calcitonin	2.56%
CD34	1.28%
CD68	1.28%
RE	1.28%
PSA	1.28%
EBV	1.28%

## DISCUSSION

Immunohistochemistry has significantly improved the outlook for pathology tests. The possibility of obtaining clarification on once doubtful and hard-to-diagnose tumors turned this technique into a powerful addition to the roster of routine test protocols.

Epithelial tissues can be immunolabeled by cytokeratins, proteins present in the cytoskeleton of epithelial cells. There are more than 25 types of cytokeratin divided into low and high molecular weight subtypes. Specific tissue types are labeled by specific cytokeratins. Squamous epithelium is labeled by high molecular weight cytokeratins such as CK4, CK5, CK6, CK13, CK14, CK17 or by a cocktail of these cytokeratins called 34βE12. Glandular epithelium is preferentially labeled by low molecular weight cytokeratins (CK8, CK18, CK 19) or by a low molecular weight cocktail called 35β11.

In diagnostic terms, IHC in SCC has limited effectiveness in subtypes that escape more traditional forms of disease. Poorly differentiated carcinomas in which histogenesis is needed for the differential diagnosis against other tumors such as sarcoma, undifferentiated, small-cell and neuroendocrine tumors, also challenge the performance of IHC protocols. SCC is labeled by the pan-cytokeratin stain (AE1/AE3) and high molecular weight cytokeratin complex 34βE12; keratinizing SCC of the nasal cavity and paranasal sinuses, basaloid SCC, and spindle-cell SCC may require additional markers. Even when looking into or confirming the diagnosis of SCC, it is important to resort to other markers to perform proper differential diagnosis against other tumor types (mesenchymal, hematopoietic, adenocarcinomas).

Even though IHC has limited indication for head and neck SCC, recent studies have assessed its prognostic relevance. Molecular pathways are known to participate in carcinogenesis. Among the most studied are the p53, p16, cyclin D1, and PTEN pathways. The association between p53, cyclin, and p16 labeling and tumor thickness, metastasis, and response to adjuvant therapy has been analyzed. Correlations have been drawn, but no consensus has been reached for head and neck SCC^5^, ^6^, ^7^. The predictive value of therapeutic response to radiotherapy has been targeted by some authors, in an attempt to correlate the degree of response to the degree of PTEN expression by IHC^8^^,^^9^.

Significant attention has been devoted to the use of target therapies in managing tumors. The epidermal growth factor receptor (EGFR) pathway acts as an oncogene by activating cell proliferation and division. When this pathway suffers mutations or is activated, keratinocytes are stimulated to proliferate and grow. The EGFR pathway is mutated in about 30% of the cases of head and neck SCC. EGFR expression has been correlated to poorer prognosis^10^. The use of monoclonal antibodies to prevent EGFR activation has garnered significant interest. The detection of this receptor through IHC aids in the choice of therapy and use of specific medication^11^.

Tumor growth and propagation are directly linked to the degree of angiogenesis. Vascular endothelium growth factor (VEGF), described in the 1980s, is a glycoprotein that binds to specific receptors to act on the mechanism of angiogenesis. Angiogenesis is essential for tumor growth and propagation and correlates with poorer prognosis. More recently, IHC has been used in the VEGF pathway to assess patient prognosis; drugs such as bevacizumab have been used to block the VEGF pathway and attain better control over the disease^12^^,^^13^.

IHC has played a significant role in primary tumor site identification in metastatic disease. Panels labeling SCC, adenocarcinoma, thyroid carcinoma, sarcoma, lymphoma, neuroendocrine carcinoma, and melanoma can improve diagnostic accuracy and thus provide valuable input to the determination of the most effective course of therapy.

All in all, IHC is a good ancillary technique. In order to optimize its use, clear indication criteria are needed. The technique helps approach head and neck tumors, mainly in diagnosing sarcomas and lymphomas and enabling their adequate classification. Its use is limited in cases of carcinomas characterized by diagnostic doubt.

In our series, most tumors were categorized as SCC (87%), as also reported in the literature^14^^,^^15^, and did not pose significant diagnostic difficulties. IHC was used in only 10.67% of the cases of carcinoma, when further clarification was needed for diagnosis and histogenesis. The use of IHC to confirm histogenesis and tumor subtype is supported by the literature^16^^,^^17^. The number of cases in our series in which IHC protocols were applied to identify carcinomas (5.77%) suggests that immunohistochemistry has not been widely used in our practice or yielded significant impact upon changes in therapy.

IHC is a useful method to identify primary tumor sites in patients with node metastasis^18^. In our series, IHC was needed in 11.69% of the cases. Although this is not a significant number, the relevance of IHC in indicating the correct histogenesis of the neoplasm in cases of unidentified primary tumor and consequently enhancing the effectiveness of adjuvant therapy cannot be neglected.

IHC has undisputed value for other tumor types (sarcomas and lymphomas). It has been used in the determination of histogenesis and tumor subtyping, and has consequently had significant impact on the choice of therapy.

## CONCLUSION

Immunohistochemistry was used in 10.67% of the head and neck tumor cases tested with traditional pathology protocols. IHC was more frequently used in carcinomas, accounting for 5.26% of all cases. In the determination of squamous cell carcinoma, IHC protocols were used in 18.42% of the tumors. The diagnosis of squamous cell carcinoma is still done largely without the use of immunohistochemistry panels. IHC is required to confirm the diagnosis in cases of metastatic disease. It is also needed in the diagnosis and subtyping of lymphomas and subtyping of sarcomas.
